# The complete chloroplast genome of *Humulus yunnanensis* and phylogenetic analysis of the genus *Humulus*

**DOI:** 10.1080/23802359.2019.1644243

**Published:** 2019-07-22

**Authors:** Li-Zhen Ling, Shu-Dong Zhang

**Affiliations:** School of Biological Sciences and Technology, Liupanshui Normal University, Liupanshui, China

**Keywords:** Chloroplast genome, *Humulus yunnanensis*, *Humulus*, phylogenetic analysis

## Abstract

*Humulus yunnanensis* is an endemic species in Yunnan, China, which is used for beer and pharmacology industry. The phylogenetic position of this species in *Humulus* remains controversial. The complete chloroplast (cp) genome sequence of *H. yunnanensis* was reported and characterized in this study. The cp genome is 153,612 bp in length and contains a pair of inverted repeats (IRs, 29,824 bp) separated by a large (87,728 bp) and small (15,390 bp) single-copy regions. A total of 112 unique genes were predicted, including 78 protein-coding genes, 30 tRNA genes, and 4 rRNA genes. The phylogenetic analysis revealed that *H. yunnanensis* is more closely related to *H. scandens* than *H. lupulus*.

*Humulus* is a small genus of Cannabaceae native to temperate regions of Northern Hemisphere. Three recognized species, *H. lupulus*, *H. scandens,* and *H. yunnanensis* are widely used in brewing, medicinal, and pharmaceutical industries. However, the phylogenetic relationships within *Humulus* have been questioned (Yang et al. 2013; Boutain 2014). Yang’s molecular phylogenetic study based on four plastid loci (*atpB-rbcL*, *rbcL*, *rps16,* and *trnL-trnF*) indicated that *H. yunnanensis* and *H. lupulus* consisted of the sister group (Yang et al. 2013). However, based on the phylogenetic result from nuclear ribosomal DNA (ITS2) and cpDNA (*petL-psbE*), Boutain (2014) validated that *H. yunnanensis* shared a closer evolutionary history with *H. scandens* than *H. lupulus*. To further determine the phylogenetic placement of *H. yunnanensis*, we sequenced the complete chloroplast (cp) genome of *H. yunnanensis* using high-throughput sequencing technology.

The fresh leaf of *H. yunnanensis* was collected from Kunming (Yunnan, Southwest of China). Specimens (14CS9673) were deposited in the herbarium of the Kunming Institute of Botany, CAS (KUN). Total genomic DNA was extracted with a modified CTAB method (Doyle and Doyle 1987). Illumina paired-end library was constructed and sequenced using the Illumina HiSeq 2500 (Illumina, CA, USA) at Novogene (Beijing, China). Approximately, 2 Gb raw data were generated. The assembly of the complete cp genome was accomplished using SPAdes (Bankevich et al. 2012). All genes encoding proteins, transfer RNAs (tRNAs), and ribosomal RNAs (rRNAs) were automatically annotated using Dual Organellar Genome Annotator (DOGMA) (Wyman et al. 2004) coupled with manual corrections.

The complete cp genome of *H. yunnanensis* (GenBank accession number: MK423880) is 153,612 bp in length and has a typical quadripartite structure. It comprises a large single-copy region (LSC, 83,697 bp) and a small single-copy region (SSC, 17,677 bp) separated by a pair of inverted repeats (IRs, 26,119 bp each). The overall GC content of the cp genome is 36.9%, while that of IRs (42.5%) is higher than that of LSC (34.6%) and SSC (30.8%) regions. The *H. yunnanensis* cp genome encodes 112 unique genes including 78 protein-coding genes, 30 tRNA, and 4 rRNA genes. There are 19 intron-containing genes, in which 10 protein-coding (*rps16*, *rpl16*, *rpl2*, *rps12*, *rpoC1*, *ndhA*, *ndhB*, *petB, petD,* and *atpF*) and 6 tRNA (*trnA-UGC*, *trnG-UCC*, *trnI-GAU*, *trnK-UUU*, *trnL-UAA,* and *trnV-UAC*) genes has a single intron and 3 genes (*clpP*, *rps12,* and *ycf3*) has two introns.

In this study, the phylogenetic analysis was performed with the newly sequenced cp genome of *H. yunnanensis* and two previously released cp genomes of *H. scandens* and *H. lupulus*. Eleven species of other five genera in Cannabaceae were used as outgroups. The identical maximum likelihood (ML) and Bayesian phylogenetic trees indicated that three species from the genus *Humulus* formed a monophyletic clade with 100% bootstrap and 1.0 posterior probability support, respectively (Figure 1). Moreover, *H. yunnanensis* was supported more closely related to *H. scandens* than *H. lupulus* (Figure 1).

**Figure 1.**
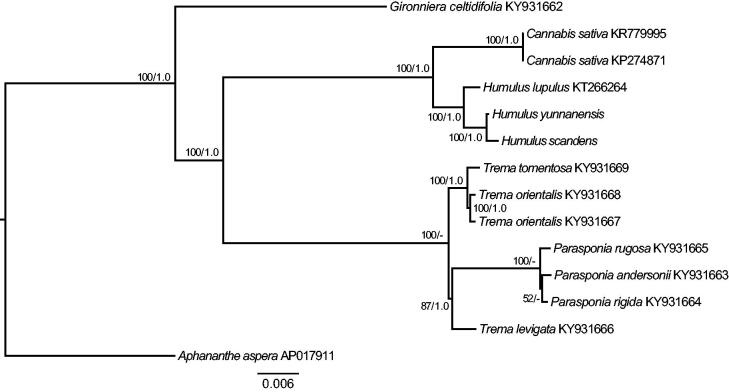
The maximum likelihood (ML) tree of *Humulus* inferred from the complete chloroplast genome sequences. Numbers at nodes correspond to ML bootstrap percentages (1,000 replicates) and Bayesian inference (BI) posterior probabilities.
